# The influence of statin-fibrate combination therapy on lipids profile and apolipoprotein A5 in patients with acute coronary syndrome

**DOI:** 10.1186/1476-511X-12-133

**Published:** 2013-09-09

**Authors:** Xiang-ping Li, Hai-rong Gong, Xian-sheng Huang, Wen-yu Huang, Shui-ping Zhao

**Affiliations:** 1 Department of Cardiology, The Second Xiang Ya Hospital, Central South University, No. 139, Renmin Zhonglu, Changsha, Hunan 410011, China

**Keywords:** Apolipoprotein A5, Triglyceride, Statin, Fibrate, Acute coronary syndrome

## Abstract

**Background:**

Statin-fibrate combination therapy has been used to treat patients with acute coronary syndrome (ACS) complicated by elevated triglycerides (TG) and decreased high density lipoprotein cholesterol (HDL-C). The purpose of this study was to evaluate the influence of the combination therapy on lipids profile and apolipoprotein A5 (apoA5) level in patients with ACS.

**Methods:**

One hundred and four patients with ACS were recruited and randomly assigned into two groups: one was statin group (n = 52), given atorvastatin (20 mg QN) or other statins with equivalent dosages; the other was combination group (n = 52), given the same dose of statin plus bezafibrate (200 mg BID). Follow-up visits were scheduled at the end of 6 and 12 weeks post treatment. Serum apoA5 levels were determined using a commercial available ELISA kit.

**Results:**

(1) Compared with that of statin monotherapy, statin-bezafibrate combination treatment not only resulted in a significant reduction of TG, TC and LDL-C levels, (all *p* < 0.05), but also led to increases in HDL-C and apoA5 levels (*p* < 0.05).

(2) The percentage changes of TC, TG, LDL-C and apoA5 levels in both groups were even bigger at 12 weeks after treatment than that at 6 weeks (all *p* < 0.05). Similarly, the rates of achieving lipid-control target were higher in statin-bezafibrate combination treatment group than those in statin monotherapy group (all *p* < 0.05).

(3) Spearman rank correlation analysis showed that the pre-treatment apoA5 level was positively correlated with TG (*r* = 0.359, *p =* 0.009). However, a negative correlation was observed between apoA5 and TG (*r* = -0.329, *p* = 0.017) after 12 weeks treatment.

**Conclusions:**

Statin and fibrate combination therapy is more effective than statin alone in achieving a comprehensive lipid control for ACS patients. Serum apoA5 elevation after statin and fibrate combination treatment could be due to the synergistic effect of both drugs on hypertriglyceridemia control.

## Introduction

Despite using intensive statin therapy, about 65% of coronary events can not be prevented in patients with coronary heart diseases. It was believed that the residual cardiovascular risk was partly associated with atherogenic dyslipidemia, including elevated triglycerides (TG) and lower high-density lipoprotein cholesterol (HDL-C) [1]. The atherogenic dyslipidemia is likely to be controlled by the statin-fibrate combination therapy [2], since fibrates have more obvious effects on elevated TG and decreased HDL-C. Interestingly, the combination therapy would have clinical significance in the setting of acute coronary syndrome (ACS), which is often characterized by higher incidence of elevated TG and decreased HDL-C than stable coronary heart disease [3]. Recent data have shown that the elevation of plasma TG in ACS patients is associated with apolipoprotein A5 (apoA5), a manifest factor indicating triglyceride metabolism [4,5]. It is noted that accumulated evidences have demonstrated that both statins and fibrates are involved in apoA5 gene expression [6-10].

Therefore, the present study was to observe the effects of statin and fibrate combination treatment on serum lipids and apoA5 levels in patients with ACS.

## Methods

### Subjects and study design

The study recruited 104 ACS patients (66 men, 38 women, mean age of 59.9 ± 7.0 years) in our hospital. Diagnostic criteria for unstable angina (UA) and acute myocardial infarction (AMI) respectively referred to the related guidelines of American College of Cardiology/American Heart Association published in 2007 (For details, please see: ①http://circ.ahajournals.org/cgi/content/full/117/2/296; and ② http://circ.ahajournals.org/content/116/7/803). Briefly, the patients were diagnosed as ACS if they had acute chest pain and changes in consecutive ECG records, with (AMI) or without (UA) changes of cardiac biomarkers such as creatine kinase (CK), CK-MB and/or troponins. Furthermore, the enrolled subjects should have their TG ≥1.70 mmol/L, LDL-C ≥2.60 mmol/L, and/or HDL-C <1.04 mmol/L. Exclusion criteria were (1) severe hepatic or/and renal diseases; (2) severe heart failure; (3) recent (<2 months) infection, immunologic disorders, thyroid diseases, recent major trauma, or cancer; (4) treatment with immunosuppressive or antiinflammatory agents; and (5) recent treatment with lipid-lowering drugs. Since the combination of statin and bezafibrate could increase the risk of myopathy or even rhabdomyolysis, subjects who had been previously diagnosed with myopathy or had high risk of these diseases were also excluded.

All patients enrolled were randomized within 48 h of hospitalization and before percutaneous coronary intervention (PCI) into two groups: (1) statin group, treated with atorvastatin 20 mg QN (or other statins in equivalent dosage, such as rosuvastatin 10 mg QN); (2) combination group, treated with the same dosage of above-mentioned statins and bezafibrate 200 mg BID. Also, according to the related guidelines of American College of Cardiology/American Heart Association, all subjects were given optimal medical therapy including aspirin, clopidogrel, ACEI/ARB, beta receptor inhibitor, and/or PCI. Follow-up visits were scheduled at the end of 6 and 12 weeks after treatment. Our study carried out on humans was in compliance with the Helsinki Declaration and approved by the Ethics Committee of the Second Xiangya Hospital of Central South University.

### Clinical and laboratory measurements

Serum samples were obtained at randomization and the end of 6 and 12 weeks thereafter. Follow-up assessments included laboratory tests, pill counts, and structured interviews assessing outcomes and potential adverse events. The serum levels of lipids, high-sensitivity C-reactive protein (hs-CRP), and fasting blood glucose, as well as hepatic and renal functions, were detected in the central laboratory of our hospital. Serum apoA5 concentration was measured by a sandwich enzyme-linked immunosorbent assay (ELISA) developed by us using monoclonal antibodies that had a lower limit of detection of 0.3 ng/ml and a linear results up to 20 ng/ml. The ELISA procedure for apoA5 detecting was performed as previously described [11].

### Statistical analysis

Descriptive statistics was used for all data analyses. For enumeration data, frequency was described and Chi-square test was used for analyses. For quantitative data analyses, mean ± SD was described. If the quantitative data were not normally distributed, rank sum test was used, while *t*-test or approximate *t*-test was used in normally distributed data. Repeated measures ANOVA analyses were performed to determine the effects of both interventions and duration of treatment. The correlation analysis was performed by Spearman rank correlation. Differences were considered significant at a value of *p* < 0.05 for all tests.

## Results

### Clinical characteristics of study subjects

The baseline clinical characteristics of subjects enrolled are summarized in Table 1. There were no significant differences between two groups in ACS type (AMI/UA), gender, age, body mass index, blood glucose, blood pressure, alanine aminotransferase, creatinine, and creatine kinase. Besides, no significant differences were observed between two groups in serum levels of lipids (including TG, total cholesterol, LDL-C and HDL-C), apoA5, and hs-CRP.

**Table 1:** Baseline clinical characteristics

	**Statin group (n = 52)**	**Combination group (n = 52)**	***p***
AMI/ UA (n)	27/25	30/22	0.144
Gender (male/female)	16/36	20/32	0.101
Age (years)	60 ± 6	59 ± 8	0.356
Body mass index (kg/m^2^)	23.48 ± 1.67	23.94 ± 2.09	0.233
Systolic blood pressure (mmHg)	131 ± 11	133 ± 12	0.195
Diastolic blood pressure (mmHg)	76 ± 8	75 ± 9	0.471
Fasting blood glucose (mmol/L)	5.45 ± 0.66	5.62 ± 0.95	0.263
ALT (u/L)	31.57 ± 21.06	29.08 ± 16.67	0.688
Cr (μmol/L)	87.4 ± 21.67	86.03 ± 18.84	0.728
CK (u/L)	86.01 ± 22.91	87.13 ± 46.38	0.875
TG (mmol/L)	1.97 ± 1.02	1.91 ± 0.87	0.740
TC (mmol/L)	4.85 ± 0.90	4.92 ± 0.83	0.644
LDL-C (mmol/L)	3.00 ± 0.43	3.04 ± 0.46	0.686
HDL-C (mmol/L)	0.89 ± 0.22	0.93 ± 0.14	0.317
Apo A5 (ng/ml)	49.49 ± 1.59	49.50 ± 2.48	0.980
hs-CRP (mg/L)	3.97 ± 3.52	3.89 ± 2.96	0.904

Note: *AMI* acute myocardial infarction, *UA* unstable angina, *ALT* alanine aminotransferase, *Cr* creatinine, *CK* creatine kinase, *TG* triglycerides, *TC* total cholesterol, *LDL-C* low-density lipoprotein cholesterol, *HDL-C* high-density lipoprotein cholesterol, *ApoA5* apolipoprotein A5, *hs-CRP* high-sensitivity C-reactive protein.

### Effects of two treatments on lipids, apoA5 and hs-CRP

At the end of study, both groups experienced significant reductions in TG, TC, LDL-C, and hs-CRP, and increases in HDL-C levels from the baseline. Compared with statin group, the statin-bezafibrate combination treatment group resulted insignificantly greater reductions in TG, TC, and LDL-C, and more increase in HDL-C (all *p* < 0.05). No significant difference was observed in hs-CRP between two groups. Both groups witnessed significantly greater changes in lipids levels after 12 weeks than 6 weeks (all *p* < 0.05), but the hs-CRP levels were similar at the two follow-up time points. Of note, the apoA5 levels increased in two groups, with a significantly greater elevation in the combination group (*p* < 0.05). Moreover, further increases in apoA5 levels in both groups were shown after 12 weeks (*p* < 0.05) (Table 2).

**Table 2:** Changes of serum lipids, apoA5 and hs-CRP levels in two groups

	**Baseline**		**After 6 weeks**		**After 12 weeks**	
	**Statin group**	**Combination group**	**Statin group**	**Combination group**	**Statin group**	**Combination group**
TC (mmol/L)	4.85 ± 0.90	4.92 ± 0.83	4.22 ± 0.88*	3.75 ± 0.65 *	4.12 ± 0.76†	3.39 ± 0.30†§
TG (mmol/L)	1.97 ± 1.02	1.91 ± 0.87	1.74 ± 0.71*	1.24 ± 0.54 *	1.62 ± 0.63†	1.15 ± 0.48†§
LDL-C (mmol/L)	3.00 ± 0.43	3.04 ± 0.46	2.30 ± 0.53*	2.06 ± 0.28 *	2.21 ± 0.55†	1.93 ± 0.29†§
HDL-C (mmol/L)	0.89 ± 0.22	0.93 ± 0.14	1.00 ± 0.26*	1.10 ± 0.13 *	1.05 ± 0.20†	1.14 ± 0.08†§
ApoA5 (ng/ml)	49.49 ± 1.59	49.50 ± 2.48	61.48 ± 6.00*	66.03 ± 8.79*	67.4 ± 5.50†	73.2 ± 6.67†§
Hs-CRP (mg/L)	3.97 ± 3.52	3.89 ± 2.96	2.01 ± 0.94*	1.61 ± 0.32*	2.03 ± 1.54*	1.62 ± 0.63*

Note: *TC* total cholesterol, *TG* triglycerides, *LDL-C* low-density lipoprotein cholesterol, *HDL-C* high-density lipoprotein cholesterol, *ApoA5* apolipoprotein A5, *hs-CRP* high-sensitivity C-reactive protein.

* *p* < 0.05, compared with baseline in the same group;

† *p* < 0.05, compared with 6 weeks in the same group;

§ *p* < 0.05, combination group versus statin group by repeated measures ANOVA analyses.

### Comparison of lipid-control efficacy of two treatments after 12 weeks

**Figure 1:**
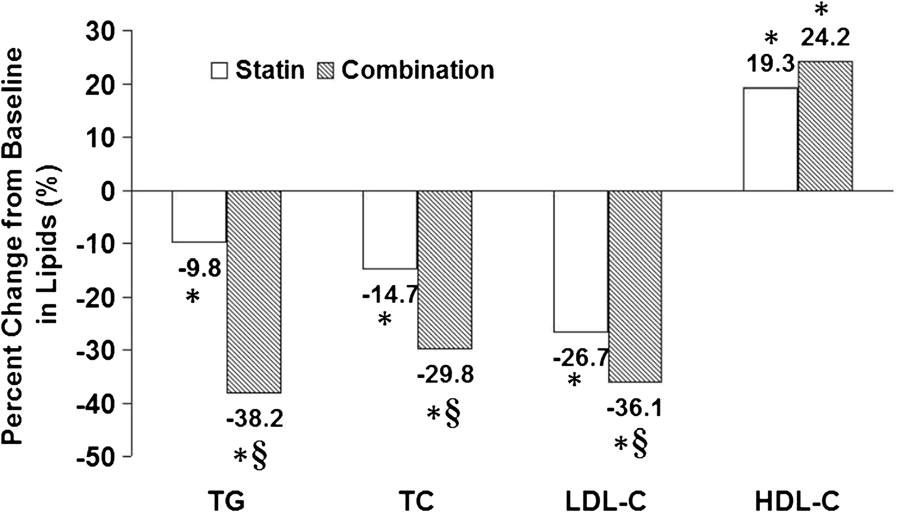
**Percentage change from baseline in lipids.** * p < 0.05, 12 week versus baseline in the same group; § p < 0.05, combination group versus statin group.

In order to compare the lipid-control efficacy of the two treatments, two endpoints of this study had been considered, including the percentage change in lipids levels and the percentage of patients achieving the therapeutic goal of dyslipidemia. Both groups had significant percentage changes in lipids levels after 12 weeks from baseline (all *p* < 0.05). As compared with the statin group, significantly greater reductions in TC, TG, and LDL-C levels were observed in the combination group (all *p* < 0.05). However, the percentage changes in HDL-C levels had no significant difference between two groups (*p* > 0.05) (Figure 1).

**Figure 2:**
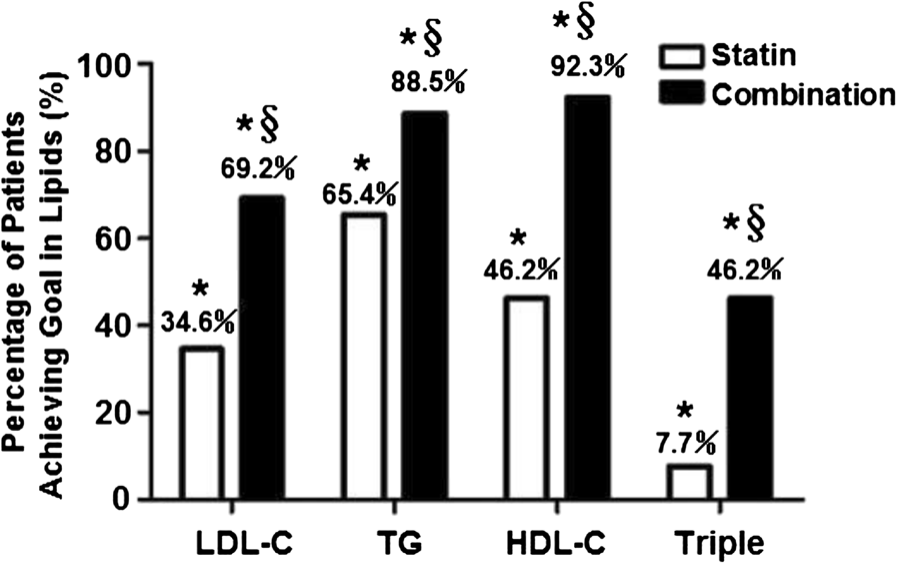
**Percentage of patients achieving therapeutic goal in lipids.** * p < 0.05. 12 week versus baseline in the group; § p < 0.05, combination group versus statin group. Triple, three lipids biomarker including LDL-C,TG and HDL-C.

According to 2007 guidelines for the prevention and treatment of dyslipidemia in Chinese adults, the percentages of ACS patients achieving the following lipid-control target values were calculated: ①LDL-C <2.07 mmol/L (80 mg/dL); ②TG <1.70 mmol/L (150 mg/dL); ③ HDL-C ≥1.04 mmol/L (40 mg/dL). After 12 weeks of treatment, the percentages of patients achieving the target levels of LDL-C, TG, and HDL-C were higher in the combination group than in the statin group (all *p* < 0.05). Of note, the percentage of patients achieving the triple lipids targets (simultaneously achieving the target levels of LDL-C,TG and HDL-C) in the combination group was much higher than that in the statin group (7.7% versus 46.2%, *p* < 0.05) (Figure 2).

### Correlation of apoA5 with other parameters before and after treatment

Before treatment, Spearman rank correlation analysis conducted in all subjects showed a significant positive correlation between apoA5 and TG (*r* = 0.359, *p =* 0.009). However, there was no evidence of any association between apoA5 and other variables such as TC, LDL-C, HDL-C and hs-CRP. After 12-week treatment, significant negative correlations of apoA5 with TG (*r* = -0.329, *p* = 0.017), TC (*r* = -0.394, *p* = 0.004) and LDL-C (*r* = -0.302, *p* = 0.024) were observed. But neither HDL-C nor hs-CRP was found to be significantly correlated with apoA5 (Table 3).

**Table 3:** Pre- and post-treatment correlation of apoA5 with other parameters

	**Correlation coefficient ( *r *)**	***p***
Pre-treatment		
TC	0.202	0.236
TG	0.359	0.009
LDL-C	0.212	0.131
HDL-C	0.114	0.834
hs-CRP	0.110	0.438
Post-treatment		
TC	-0.394	0.004
TG	-0.329	0.017
LDL-C	-0.302	0.024
HDL-C	0.205	0.114
hs-CRP	-0.051	0.717

Note: *TC* total cholesterol, *TG* triglycerides, *LDL-C* low-density lipoprotein cholesterol, *HDL-C* high-density lipoprotein cholesterol, *ApoA5* apolipoprotein A5, *hs-CRP* high-sensitivity C-reactive protein.

### Adverse events

During the whole study period, there were no severe adverse events reported, such as severe myalgia, liver damage, renal failure, or rhabdomyolysis. No discontinuation of the study medication occurred due to adverse events. No significantly higher incidence of adverse events was observed in the combination group than in the statin group. Furthermore, both treatment groups resulted in no significant elevation in serum levels of ALT, CK and Cr (Table 4).

**Table 4:** Pre- and post-treatment serum levels of ALT, CK and Cr

	**Before treatment**		**After 12-week treatment**	
	**Statin group**	**Combination group**	**Statin group**	**Combination group**
CK (u/L)	86.01 ± 22.91	87.13 ± 46.38	94.43 ± 27.06	93.63 ± 33.62
ALT (u/L)	31.57 ± 1.06	29.08 ± 16.67	29.75 ± 12.92	31.39 ± 12.49
Cr (μmol/L)	87.40 ± 21.67	86.03 ± 18.84	83.60 ± 18.10	82.83 ± 10.47

Note: *CK* creatine kinase, *ALT* alanine aminotransferase, *Cr* creatinine.

## Discussion

Dyslipidemia is an established risk factor for the development of coronary heart disease. Although statin therapy is the current recommended primary treatment for dyslipidemia, many patients fail to reach adequate lipid-control goals and remain at a significantly increased risk of cardiovascular events [1,2]. Given this residual risk, there is a critical need for additional lipid-control therapies that could augment the efficacy of statins to further lower the burden of residual atherogenic dyslipidemia including elevated TG and low HDL-C level. Statin-fibrate combination therapy has much clinical significance for ACS patients with elevated TG and decreased HDL-C [3]. In the present study, we demonstrated that the statin-fibrate combination therapy resulted in greater reductions of TG, TC and LDL-C (all *p* < 0.05) and more increase of HDL-C in ACS patients (*p* < 0.05). Moreover, the rate of achieving therapeutic goal of triple lipids (LDL-C,TG and HDL-C levels) in patients with combination treatment after 12 weeks was much higher than that with statin treatment (7.7% versus 46.2%, *p* < 0.05, Figure 2). Thus, our results suggest that the combination of statin and fibrate has synergetic effect of lipid regulation which contributes to improve statin-associated residual dyslipidemia, especially in ACS patients with hypertriglyceridemia and/or low HDL-C.

The present study further determined the role of apoA5 in triglyceride-lowering effect by statin-fibrate combination. We found that both treatments contributed to the elevation of apoA5 level, with greater elevation noted in the combination treatment. Interestingly, a positive correlation has been observed between apoA5 and TG before treatment. However, after 12-week treatment, a negative correlation between apoA5 and TG was noted. Evidences from genetically engineered mice studies have shown that apoA5 is a potential factor of plasma TG reduction. Plasma TG level increased four-fold in endogenous apoA5 gene deficiency mice and decreased by 65% on expression of human apoA5 gene [4]. Similarly, inherited apoA5 gene deficiency is associated with severe hypertriglyceridemia in human [12]. However, contrary to expectations based on studies in individuals bearing nonsense apoA5 gene mutations and studies in genetically engineered mice, the results from the Epic-Norfolk Population Study showed that plasma apoA5 level was positively correlated with plasma TG [13]. Besides, a positive correlation between apoA5 and TG was also observed in patients with type 2 diabetes [10]. Our previous study in ACS patients had also observed such positive correlation before lipid-lowering treatment [14], but the mechanism is quite unclear. We presume that plasma apoA5 level may have compensatory increase and the effect of apoA5 on reducing TG level may be impaired during acute inflammation or stress state. Together, these findings from human studies indicate a complex relationship between plasma apoA5 and TG in humans. Nonetheless, our findings in this study implicated a role of apoA5 in plasma TG reduction by statins and/or fibrates. It is well documented that fibrates, agonist of PPARα, result in plasma TG reduction by upregulating apoA5 expression. A PPAR response element has been identified at the transcription start site of apoA5 gene, whereby pharmacological PPAR agonists (such as fibrates) may exert their beneficial hypotriglyceridemic actions [6,7]. Our previous findings indicated that statin and fibrate synergistically increased apoA5 and decreased TG by upregulating PPARα in rats [9]. Therefore, we conclude that statin and fibrate can synergistically increase plasma apoA5 level, which results in more hypotriglyceridemic effects.

Atherosclerosis is an inflammatory disease and acute inflammation is a major factor underlying the development of ACS [15]. In the acute inflammatory setting of ACS, lipid and lipoprotein metabolism experiences systemic alteration including elevated TG and decreased HDL-C [16]. Furthermore, apoA5 gene is regulated by proinflammatory cytokines and apoA5 may be involved in inflammation-associated hypertriglyceridemia [17,18]. Therefore, we examined the potential cross-talk between inflammation and apoA5 in ACS. Our findings demonstrated that both treatments could lead to the reduction of hs-CRP, an inflammatory biomarker. This suggests that both lipid-lowering agents can inhibit inflammatory reaction, and contribute to lower incidence of cardiovascular events [19]. However, no significant correlation was found between apoA5 and hs-CRP in our present study. Our findings indicated that elevation of serum apoA5 level by statin or fibrate could be independent from their anti-inflammatory effect.

In general, statin and fibrate combination therapy is more effective than statin monotherapy in achieving a comprehensive lipid control target for ACS patients. Furthermore, our data suggest that the synergy of the combination therapy in reducing triglyceride may be involved with the elevation of apoA5 level.

## Consent

Written informed consent was obtained from the patient for the publication of this report and any accompanying images.

## Competing interests

The authors declare that they have no competing interests.

## Authors’ contributions

XPL carried out the design of the study, the patient's follow-up, the data analysis and drafted the manuscript. HRG carried out biochemical detection and participated in the patient's follow-up, the data analysis and in drafting the manuscript. XSH participated in following up the subjects and in drafting the manuscript. WY H carried out biochemical detection and participated in drafting the manuscript. SPZ conceived of the study and provided comments on the manuscript. All authors read and approved the final manuscript.
